# 

**Published:** 1882-12

**Authors:** 


					﻿VOL. IL
DECEMBER, 1882.
NO. 12.
THE
fiOMlEOPATHlC pHY^lClAfl,
A MONTHLY JOURNAL OF MEDICAL SCIENCE.
'•'■Magna est veritas, et prcEvalebit.” ■
EL CT, XjEjZElL HVT. ID.,
EDITOR.
CONTENTS:
- (See inside of Cover.)
PHILADELPHIA:
No. 3109 CHESTNUT STREET.
ND'W YORK.
A. L. CHATTERTON PUBLISHING COMPANY, PUBLISHER’S AGENTS.
X.O3ST DO KT :
* ALFRED HEATH <fc CO., Sole Agents for Great Britain,
114 Ebury Street, Eaton Square.
Hearty Subscription, $2.50, invariably in advance.
Entered at the Ph iladelphia Post-Office as Second Class Mail Matter.
				

